# Saccade Landing Point Prediction Based on Fine-Grained Learning Method

**DOI:** 10.1109/access.2021.3070511

**Published:** 2021-04-01

**Authors:** AYTHAMI MORALES, FRANCISCO M. COSTELA, RUSSELL L. WOODS

**Affiliations:** 1 BiDA-Lab, Department of Electrical Engineering, Universidad Autonoma de Madrid, 28049 Madrid, Spain; 2 Schepens Eye Research Institute, Massachusetts Eye and Ear, Boston, MA 02114, USA; 3 Department of Ophthalmology, Harvard Medical School, Boston, MA 02115, USA

**Keywords:** Saccade, eye movement, gaze-contingent, recurrent neural networks, LSTM, fine-grained learning

## Abstract

The landing point of a saccade defines the new fixation region, the new region of interest. We asked whether it was possible to predict the saccade landing point early in this very fast eye movement. This work proposes a new algorithm based on LSTM networks and a fine-grained loss function for saccade landing point prediction in real-world scenarios. Predicting the landing point is a critical milestone toward reducing the problems caused by display-update latency in gaze-contingent systems that make real-time changes in the display based on eye tracking. Saccadic eye movements are some of the fastest human neuro-motor activities with angular velocities of up to 1,000°/s. We present a comprehensive analysis of the performance of our method using a database with almost 220,000 saccades from 75 participants captured during natural viewing of videos. We include a comparison with state-of-the-art saccade landing point prediction algorithms. The results obtained using our proposed method outperformed existing approaches with improvements of up to 50% error reduction. Finally, we analyzed some factors that affected prediction errors including duration, length, age, and user intrinsic characteristics.

## I. INTRODUCTION

**FIGURE 1.**
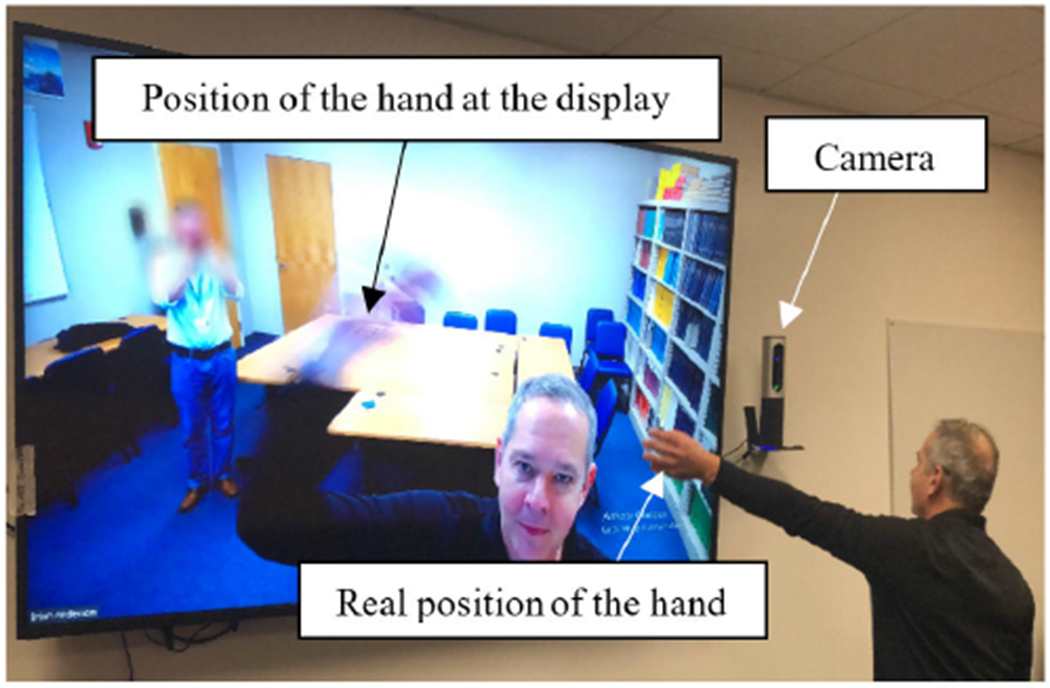
An illustration of system latency in which the virtual arm (on display) is shown in an earlier location of the real arm as the arm was extended. The large television (Vizio M80-D3) was running at 60Hz frame rate. The system latency was about 30ms, which is low for a digital display [10].

Gaze-contingent displays [1] have been used in video streaming [2], [3], robot-assisted surgery [4], human-computer interfaces for new virtual reality environments [5], reading research [6] and simulation of impaired vision [7], [8], among others. These systems allow researchers to investigate a variety of visual phenomena, including eye movement guidance in reading, stability of vision, visual search strategies, and scene perception. Due to data transmission, image processing, and data display preparation, all systems have an updating latency of at least 10 ms [9] to 12 ms [10] that introduces an updating error (see Figure 1).

Saccades are very fast eye movements made between phases of fixation which bring the image of the region of interest to the fovea, the small (1 to 2°) retinal region with the highest resolution. During typical viewing, we make about three saccades per second. Saccades are one of the fastest muscle movement, up to 1,000°/s (peak angular velocity). Saccades have short durations, lasting about 15 to 200ms, with the duration closely tied to the length (amplitude) and to the peak velocity. While the common perception is that saccades are ballistic eye movements that are not modified during flight and have a straight path, this is often not the case [11]. Since saccades are fast, due to the unavoidable latency of gaze-contingent systems (that are intended to update displays according to gaze location), the gaze is no longer at the measured location by the time the display can be updated.

Saccades are responses of the visual system mainly to visual stimuli. One approach to predicting the next saccade landing point is to analyze the visual scene for the most likely new region of interest. These visual salience models have improved over recent decades [12]. The computations for visual salience may be challenging for a real-time application and a scene that is in motion. Another limitation of the visual salience approach is that such models have not been shown to be able to predict the timing of saccade initiation, so could not inform the timing of the change of a gaze-contingent display. As an alternative approach, we propose using the early part of a tracked saccade to predict the landing point. Such an approach is agnostic to visual stimuli, employing saccade dynamics alone.

Modelling saccade dynamics is challenging, as saccades may not be ballistic or straight [11]. The relationship between saccade amplitude and maximum velocity (the saccadic main sequence) was first described by Bahill *et al.* [13]. Longer saccades have been modeled with lognormal functions, while Gaussian functions [14], or compressed exponential functions [15] may be better for shorter saccades. Various models have been proposed or used to predict saccade amplitude, direction or trajectory, including mirroring the data points before the peak velocity [14], the main sequence [16], [17], Kalman filters [18]–[20], a compressed exponential function [15], a Taylor series [21], and a skewed Gaussian function [22]. Saccade prediction could improve the experience with gaze-contingent displays [15], [18]–[21].

Each saccade landing point determines the new fixation or gaze location, with that object being imaged at the fovea. Prediction of landing point may reduce problems due to the system-update latency of gaze-contingent displays, as the display could be updated at the predicted saccade landing point while the saccade was in flight [22]–[26]. Due to the speed of saccades, vision is very poor during the saccade [27], so an update is unlikely to be noticed. Previous approaches to predicting saccade landing location include polynomial fitting [24], Recurrent Neural Networks (RNNs) [25], and velocity profile fitting [22]. Here we present a new method for landing-point prediction.

**FIGURE 2.**
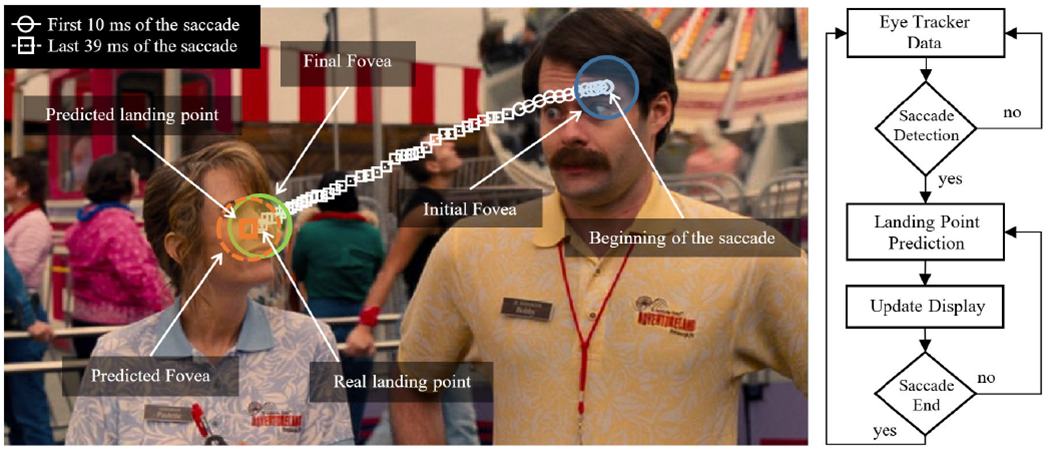
Landing point prediction from a 10 ms sequence (small white circles) from a real saccade movement acquired with an EyeLink 1000 eye tracker (1,000Hz). The trajectory of the 49ms saccade is represented by the Cartesian coordinates of each of the samples obtained from the eye tracker (small white circles and squares). The gaze positions are in the region of interest of the viewer, with the fovea represented by the colored circles, each with a diameter of 2.5°. Note that prediction is performed independently of the stimuli (i.e. video frames are not used in the prediction).

**FIGURE 3.**
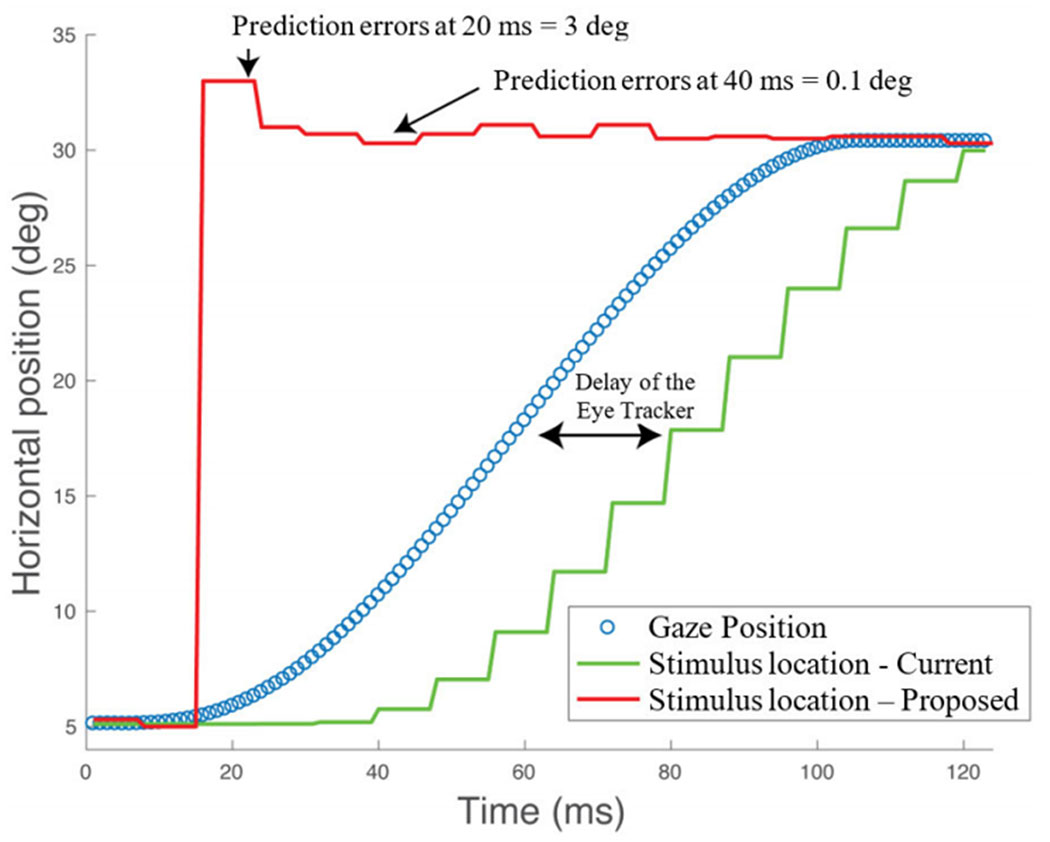
Gaze position (blue) during one left-to-right saccade. Updating of a gaze-contingent stimulus is shown for simple updating which was delayed by the system latency (green line) on an iMac Intel Core i7 (60 Hz display) connected to an Eyelink 1000. The red line used our proposed method that predicted the landing point of the saccade and moved the stimulus to the predicted landing location so that it was visible immediately on saccade landing. The approach assumes that the stimulus is not visible during a saccade (“saccadic suppression”).

Figure 2 shows an example of a measured saccade path captured during natural viewing of a video clip. The figure shows the path when the region of interest changed from stimulus A (the man) to stimulus B (the woman). In saccade landing point prediction based on eye tracker data, the prediction is performed every time a saccade starts. The goal is to predict the target gaze position (defined by the landing point of the saccade) using the first coordinates provided by the eye tracker. The prediction is performed using exclusively the eye tracker data (i.e. the stimuli are unknown). The landing point prediction can be updated with every new position provided by the eye tracker. The figure illustrates some of the main challenges associated with saccade-landing-point prediction including: i) nonlinear trajectory of the movement; ii) measurement noise of the eye tracker; and iii) nonlinear velocity profiles (e.g. the distance between data samples during the first 10ms and the last 38ms varies). Figure 3 illustrates the target gaze position (horizontal axis only), the delay introduced by the hardware, and the prediction of the landing point (horizontal coordinate) obtained with the proposed prediction updated every 10 ms.

Among the disruptive technologies of last years, Deep Learning (DL) has become a thriving topic [28]. The increasing volumes of available data and the increased computational power are used to model complex problems in terms of a hierarchy of simpler processing units. Data-driven learning methods has allowed advances in many fields such as Computer Vision [29], Speech Recognition [30], and Natural Language Processing [31].

Recurrent Neural Networks (RNN) are architectures especially useful when modeling sequences [32]. The number of applications of RNNs is large and it includes modeling sequences of data with very different natures such as handwritten-signature recognition [33], [34], audio processing [30], [35], and epilepsy [36]. RNN architectures incorporate dynamic temporal relationships by self-connected units (i.e. recurrent connectivity). This connectivity serves to incorporate temporal context into the learning process. The context can be passed through units or stored in specific memory units as an internal state. However, traditional RNN are not capable of maintaining this contextual information for long term temporal relationships. This problem is known as vanishing gradient problem [37]. Long Short-Term Memory (LSTM) [38] was proposed trying to solve the vanishing problem. LSTM networks incorporate specific memory units to model long-term temporal relationships.

A loss function is used to guide the learning process of data-driven approaches. Choosing the loss function that best fits the modelling task at hand is critical. The number of loss functions in the literature is large. Among the different learning strategies, fine-grained learning is aimed to distinguishing sub-categories inside a primary category. Fine-grained learning merges sub-ordinate patterns allowing improved classification [39] and regression models [40].

The main contributions of our work are:

- A new saccade landing point prediction method based on LSTMs and a fine-grained learning strategy.
- A comprehensive analysis of the performance over more than 200,000 saccades from 75 participants acquired during viewing video. This analysis includes the effects of covariates including user-dependent (e.g. neuromotor physiognomy) and task-dependent (e.g. number of samples available to make the prediction, length of the saccade) characteristics.
- Comparison with state-of-the-art approaches [24], [25]. The experiments demonstrate the superior performance of the fine-grained learning approach in comparison with traditional learning functions. The results obtained using our proposed method outperformed existing approaches with improvements ranging from 20% to 50% error reduction.

The rest of this paper is organized as follows: the end of Section 1 presents the related works. Section 2 describes the saccade landing point prediction approach, the learning strategy, and the database used for the experimental protocol. Section 3 reports the experiments and results. Finally, Section 4 summarizes the conclusions.

### A. RELATED WORKS

There have been previous efforts on predicting the saccade landing point from eye tracker data [22], [24]–[26]. The method used in [24] was based on polynomial fitting trained to predict displacement and angle of the saccade. The results reported in [24] suggested that it is possible to predict the landing point of saccades and its potential to improve gazerendering systems. The method in [22] was based on velocity profile prediction, using the close relationship between velocity and amplitude [13]. They proposed fitting a Gaussian velocity function to predict the amplitude of the saccade. Both studies [24], [22] used eye-movement data obtained from non-natural viewing conditions, with saccades made to pre-defined visual stimuli. The prediction of saccade landing point using RNNs was proposed in [25], [26] where researchers evaluated Long Short-Term Memory Networks (LSTM). The results in [25] demonstrated the capacity of these networks to model the saccade movement and outperform function-fitting approaches in natural viewing. These approaches try to exploit the predictable characteristics of the oculomotor system. The aim is modeling the movement using only the early part of the saccade.

As noted above, a potential alternative approach uses visual attention, in which the goal is to predict group behavior or typical behavior of people within a group [12]. These methods are usually aimed to model mid-term user behavior rather than individual saccade movements. Visual attention models can be divided into two categories: *i*) Static saliency maps prediction based on static fixations [41]–[44]; and *ii)* Dynamic saliency maps prediction incorporating the temporal sequence of fixations [45]–[47]. It is not clear that visual attention models will be able to provide the timing of saccade events at the individual level or to handle situations when there are two equally salient new locations (as in Figure 2) that produce bimodal gaze distributions (e.g. Figure 1 in [48]) as required for gaze-contingent and virtual-reality systems.

## II. MODELING SACCADIC EYE MOVEMENTS

### A. NATURAL VIEWING DATABASE

Data was drawn from a shared database [49]. It comprised the gaze of 75 human participants (37 female, 38 male) with normal sight who participated in studies approved by the Institutional Review Board of the Schepens Eye Research Institute and were in accordance with the Declaration of Helsinki (Code of Ethics of the World Medical Association). Participant screening included self-reported ocular health, measures of central visual function (visual acuity and contrast sensitivity), and evaluation of central retinal health and fixation stability (retinal imaging using a Nidek MP-1, Nidek Technologies, Vigonza, Italy or an Optos OCT/SLO, Marlborough, MA, USA). Participants had visual acuity of 6/7.5 (0.10 logMAR) or better, letter contrast sensitivity of 1.67 log units or better, no evidence of retinal defects and steady central fixation.

Participants either watched:

1. 40 to 46 of 206 thirty-second “Hollywood” video clips. Those included a variety of genres and depicted activities. The genres included nature documentaries (e.g., *Food, Inc, The March of the Penguins*), cartoons (e.g., *Cloudy with a Chance of Meatballs, The Simpsons Movie*), and dramas (e.g., *Stardust, The Stepfather).* Participants were instructed to watch each video clip “normally, as you would watch television or a movie program at home” and then to describe the contents of the clip [50], [51]. This group of 62 participants contributed 108,640 saccades.
2. two to five 30-minute movie clips (*Bambi, Flash of Genius, Inside Job, Juno*, and *Kpax*). This group of 14 participants (one was also in the first group) contributed 110,695 saccades.

A detailed description and additional information about the database can be found in [49].

**FIGURE 4.**
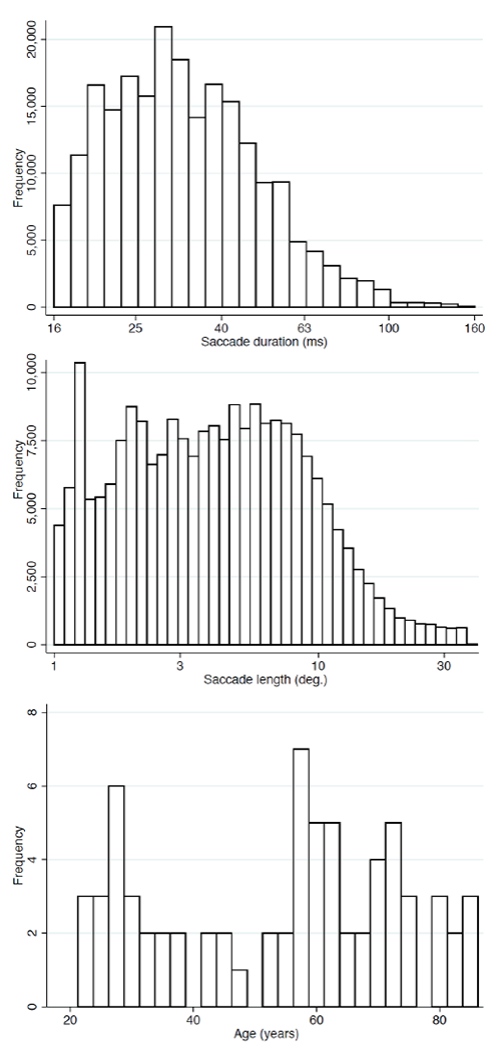
Descriptive histograms showing the duration and length (amplitude) of the 219,335 saccades, and the ages of the 75 participants.

Figure 4 presents the database statistics for duration and length of the saccades, and age of the participants. As we will see in the experimental section, these factors are important to determine the prediction error. The average duration was 34 milliseconds, while average length was 5.8 degrees. The average age of the participants in the database was 54 years old with similar number of participants younger and older than 60 years old.

### B. SACCADE DETECTION

Gaze was tracked with an EyeLink 1000 infra-red video-based eye tracker (SR Research Ltd., Mississauga, ON, Canada) at a sampling rate of 1,000 Hz while participants viewed a 27” display (60 × 34 cm) from 1 m for a 33 × 19° potential viewing area. Using the method described in [21], 219,335 saccades were detected off-line. Raw data^1^ were used to develop and test the algorithms. EyeLink’s online data parser was used to identify and remove blinks. Periods surrounding the missing data were removed if the speed was >30°/s. Missing data for blinks were replaced by interpolation using cubic splines. Before saccade detection, the raw data was smoothed with a 3rd-order Savinsky-Golay filter with a window size of 15. This smoothing made saccade detection more reliable. Speed was calculated as the first derivative of the eye position with respect to time. Saccade commencement was signaled by speed >30°/s for at least 10 ms. Saccade completion was signaled by speed <30°/s. Saccades were restricted to: (1) <40°, as this was approximately the maximum diagonal dimension of the display; and (2) >1° in amplitude and 15 ms in duration to exclude microsaccades. Additional restrictions of an initial speed <0.075°/ms, terminal speed <0.3°/ms, and removal of saccades with a velocity at first quartile of duration <0.15 peak velocity, removed eye movements with uniform but unrealistically low velocity profiles during their initial phase, which may have been pursuit eye movements. The smoothed data of the saccades that were identified using the above procedure were then replaced with the raw data. The rationale was that a real-time algorithm would have access to raw data, thus our input was realistic.

### C. SACCADE LANDING POINT PREDICTION BASED ON FINE-GRAINED LEARNING STRATEGY

Saccades are complex neuromotor eye movements involving six muscles per eye, acting in a coordinated manner in a very short time. The aim of a saccade lading point prediction algorithm is to estimate the final foveated region using the first samples of the saccade obtained by the eye. The landing point is defined according to: (i) prediction model (LSTM Neural Network in our approach) and; (ii) input data provided by the eye tracker. The length, duration, and curvature of the sequence varied depending on the oculomotor setup of the movement. To model the non-monotonic trajectories of the saccade, we update the prediction every 5ms with new data provided by the eye tracker. The problem of the latency of gaze-contingent systems will be partially solved if the prediction is sufficiently accurate early in the saccade (e.g. first 15ms).

**FIGURE 5.**
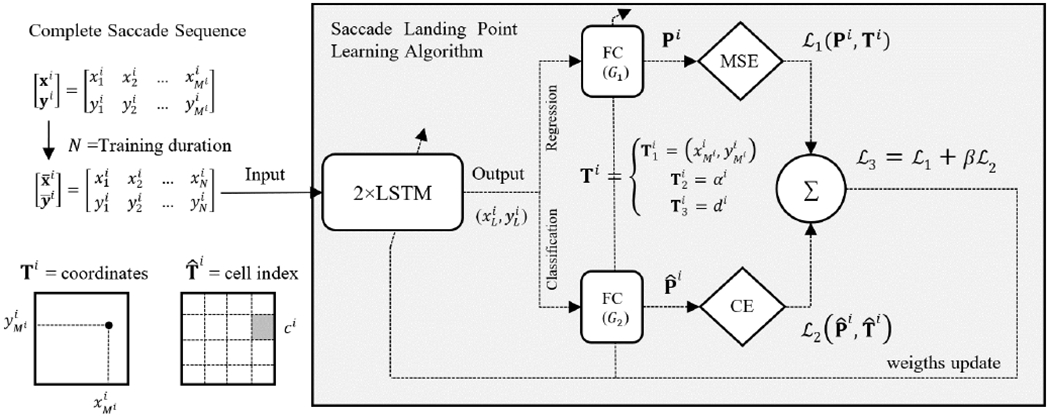
Saccade landing point learning algorithm was based on three loss functions: $L_{1}$ was obtained as the Mean Square Error (MSE) between the Prediction (P) and the Target function (T); $L_{2}$ was obtained as the Cross Entropy (CE) between the discretized Prediction ($\hat{P}$) and the discretized Target ($\hat{T}$); finally, $L_{3}$ was obtained as a linear combination of $L_{1}$ and $L_{2}$. FC = Fully connected layer.

In addition to our proposed approach, we have evaluated different learning strategies to model saccades. Figure 5 shows our learning algorithm, which can be summarized in:

#### 1) TARGET FUNCTION (T)

We evaluated two different strategies based on three Target functions (**T**_1_, **T**_2_, **T**_3_). The first strategy consisted of modelling the Cartesian coordinates of the landing point: **T**_1_ = (*x_M_*, *y_M_*). The output of the network (*x_L_*, *y_L_*) was direct prediction of the position of the landing point. The second strategy included the training of a specific network for each of the Target functions (**T**_2_, **T**_3_). The first network was trained to predict the angle/direction of the saccade movement (**T**_2_ = *α*), while the second network was trained to predict the displacement (**T**_3_ = *d*). The output of both networks was combined with the initial points (*x*_1_, *y*_1_) to obtain the landing position (*x_L_* = *x*_1_ + *d* cos *α*,*y_L_* = *y*_1_ + *d* sin *α*).

#### 2) LOSS FUNCTIONS (L)

Inspired by the learning strategy proposed in [40] we defined three loss functions. The first loss function ($L_{1}$) performed a regression loss trying to obtain a fine prediction of the landing point. The LSTM blocks were connected to a dense output layer with *G*_1_ units (*G*_1_ = 2 for **T**_1_, *G*_1_ = 1 for **T**_2–3_) and linear activation. The loss function was calculated as the *l*^2^ norm between the Target and Predicted distributions:

(1) $$L_{1}(P,T)=∥T-P∥$$

The second loss function ($L_{2}$) exploited the stability of the training procedure based on a softmax layer (categorical cross-entropy loss). The Target function (**T**) was discretized ($\hat{T}$) in *G*_2_ classes, and the loss function was calculated as:

(2) $$L_{2}\left(\hat{P},\hat{T}\right)=-\sum_{c=1}^{G_{2}}{\hat{T}}_{c}\;\log\;\left({\hat{P}}_{c}\right)$$

where *G*_2_ was the number of classes for each Target function (*G*_2_ = 64 for landing point **T**_1_, *G*_2_ = 32 for displacement and angle **T**_2–3_), log was the natural logarithmic, ${\hat{T}}_{c}$ was a discrete label associated to class *c*, and ${\hat{P}}_{c}$ was the predicted probability for the class *c*. For the discretization, the screen was divided into a 64 cells grid and each landing coordinate was replaced by the index of the nearest cell (see Figure 5). The displacement and angles were discretized into 32 equidistant values (from 0 to *π* for angle, from 0.1 to 40 degrees for displacement).

Finally, the third loss function ($L_{3}$) was calculated as a linear combination of previous ones with a weighting parameter *β* (empirically set to 0.7):

(3) $$L_{3}=L_{1}+\beta L_{2}$$

#### 3) ARCHITECTURE OF THE NETWORK

Different architectures were evaluated during the experiments. The number of layers was chosen to maximize the prediction accuracy for the minimum number of parameters possible. The final neural network architecture was based on two LSTM hidden layers, and a fully connected dense layer with a linear activation (**T**) and softmax activation ($\hat{T}$). The number of units of the output dense layer (*G_i_*) varies depending on the loss function $L_{i}$ and the target function (see loss function explanation and Figure 5).

#### 4) TRAINING PROCEDURE

As gaze was acquired at 1,000Hz, we obtained one sample every 1ms. Throughout the paper we use time units to refer to the available samples. We trained six models that varied in the time available to make the prediction. The six models were trained to predict the landing point from given sequences with only the first 10ms, 15ms, 20ms, 25ms, 30ms or 35ms. The proposed architecture was trained using the Cartesian coordinates of the training saccades, according to the following steps:

- Each of the saccade training sequences (**x**^*i*^, **y**^*i*^) with length *M^i^* were truncated with length *N* = 10, 15, 20, 25, 30, 35) depending on which of the six models was trained. Zero padding was applied when *M^i^* < *N*.
- The target function **T** was chosen depending of the output to be modeled: landing coordinates ($T_{1}=\left(x_{M}^{i},y_{M}^{i}\right)$), angle (**T**_2_ = *α^i^*), or displacement (**T**_3_ = *d^i^*).
- The loss function was chosen depending of the learning strategy: mean square error between Prediction and Target ($L_{1}$); cross entropy between discretized values of Prediction and Target ($L_{2}$); linear combination of both losses ($L_{3}$).
- The architecture of the network was built with two LSTM layers (each consisting of 32, 64, or 128 units depending of the setup evaluated, see Table 1) and a dense layer (*G* units with linear activation for **T**_1_, and softmax activation for **T**_2_ and **T**_3_). Additionally, we evaluated both forward and bidirectional learning.
- The truncated training sequences (${\overset{‒}{x}}^{i},{\overset{‒}{y}}^{i}$) and the target output **T** were used to train each of the six models (different values of *N*). All LSTMs were trained using a Backpropagation algorithm with Adam optimizer, and a maximum number of 60 epochs.

#### 5) PREDICTION OF LANDING POINTS

For a given a saccade sequence, the first prediction was provided from the first 10ms and updated every 5ms using its corresponding model and the samples available to make the prediction. The (**x**^*i*^, **y**^*i*^) coordinates of the saccade were used as input of the trained model (truncated to the nearest *N*) and the output of the network was provided as the predicted landing point ($x_{L}^{i},y_{L}^{i}$).

## III. EXPERIMENTS AND RESULTS

### A. EXPERIMENTAL PROTOCOL

The experimental protocol used to train the models was based on an open-set setup. The participants employed to train the saccade prediction models were not used to evaluate such models. The database was split into training (80% of the participants available in the dataset) and testing set (remaining 20% of the participants). These sets were chosen randomly, and the process was repeated ten times. The performances obtained from all ten experiments were averaged to provide the results. Each user (i.e. individual) contributed to the test set with a different number of samples (i.e. saccades). To avoid a biased result, the prediction error was first calculated individually for each user, and then averaged for all users in the test set.

The experiments include nine different approaches defined by the different learning strategies proposed in Section 2.2, plus three baseline approaches published in previous works [24], [25]:

- *Polynomial Fitting [*24*]:* this method was one the first approaches of saccade-landing-point prediction in the literature. The authors modelled the amplitude of the saccade as a polynomial problem. The direction was estimated from the samples available for the prediction (angle between first and last saccade sample). The training method was based on a polynomial fitting that minimized the error between the prediction and the real landing point. To guarantee a fair comparison, we applied the proposed approach to the training data available in our dataset.
- *Approach A - Feed Forward Neural Networks (FFNN) [25]:* FFNNs are the basic architecture of Neural Networks and have been demonstrated to be useful modeling nonlinear functions [52]. This approach was composed of two hidden layers (32 units and ReLu activation of each), and an output layer with two units (i.e. coordinates of the landing point). The $\left(\bar{x^{i}},\bar{y^{i}}\right)$ coordinates were used as the input of the model.
- *Approach B – RNN:* A RNNs is an architecture trained to model temporal relationships in machine learning problems. The $\left(\bar{x^{i}},\bar{y^{i}}\right)$ coordinates were used as the input of the model.
- *Approach C – LSTM and Coordinate Regression [*25*]:* A LSTM is a RNN architecture specifically designed to solve the vanishing problem using memory units. In this approach, the learning strategy was defined by the coordinate target function **T**_1_ = (*x_M_*, *y_M_*) and the regression loss function $L_{1}$.
- *Approach D - LSTM and Direction And Displacement Regression:* the learning strategy of this approach was defined by the target functions (**T**_2_ = *α*, **T**_3_ = *d*) and the regression loss function $L_{1}$.
- *Approaches E, F, G – LSTM And Fine-Grained Learning:* these approaches were defined by the coordinate target **T**_1_ = (*x_M_*, *y_M_*) and the combined loss function $L_{3}$. The difference between approaches *D* to *F* was the number of units in the LSTM layers.
- *Approach H – Bidirectional LSTM and Fine-Grained Learning:* this approach introduced bidirectional learning to approach *D*. Bidirectional networks connected hidden layers of opposite directions to the same output. These layers allowed us to model previous and next positions with more information.

For each approach, we trained six models (*N* = 10, 15, 20, 25, 30, 35) according to the protocol presented in Section III.A. All approaches were evaluated using the same evaluation set. The error was calculated as the *l*^2^ norm between the predicted and the real landing points.

### B. PREDICTION PERFORMANCE

Table 1 presents the performance obtained by the different approaches. The errors were calculated as the Euclidean distance between the real (*x_M_*, *y_M_*) and the predicted landing point (*x_L_*, *y_L_*). All errors were averaged depending on their length: i) saccades shorter than 5 degrees (column 9), ii) saccades shorter than 15 degrees (column 10) iii) saccades longer than 15 degrees (column 11), and iv) all lengths (column 12). Additionally, we report the average prediction error for all the saccades in the test set (in natural viewing, the number of small saccades is much larger than long saccades [11], and see Figure 4b). Table 1 shows the superior performance of our proposed LTSM algorithm (approaches *D* to *G*) with average improvement of more than two degrees compared to two state-of-the-art methods [24], [25]. The results show the superior performance of the learning strategy based on predicting the final landing points (approaches *E* to *H*) in comparison with the method based on direction and displacement (approach *D*). The results suggest that the network was capable of modeling the entire movement using the exclusively the initial part of the sequence. Displacement, curvature, and angles were modelled all together in this strategy. Regarding the loss function, the combined loss ($L_{3}$) outperformed the regression loss ($L_{1}$). The learning process involving both regression and cross-entropy (approaches *E* to *G*) was better than the approaches based on traditional regression functions (approaches *B* and *C*). Finally, the bidirectional learning (approach *D*) did not improve the forward learning (approach *E*). Table 1 includes the standard deviation of the errors. Additionally, Table 1 summarizes the number of parameters of the approaches evaluated in this work. While the polynomial method proposed in [24] was defined by 18 parameters, the neural network architectures were comprised of many more parameters that could be successfully trained for saccade-landing-point prediction due to the large number of saccades available in the datasets that are becoming available.

Table 1 includes the execution times for a prediction of an input saccade of ten milliseconds (*N* = 10). All the experiments were carried out in an Intel Core i7-8750H CPU @ 2.20GHz, 32 GB RAM, Nvidia GeForce RTX 2080. As was expected, the polynomial fitting had the lowest execution time. Deep learning approaches were around 5 milliseconds with small differences between approaches, except for the Bidirectional layer with an execution time of 8.8 milliseconds. Note that execution time of the deep learning approaches varied with the input size *N*. For example, the execution time for different input sizes (*N* = 10, 15, 20, 25, 30, 35) of the approach *F* were [5.2, 6.1, 7.3, 8.2, 9.4, 10.4 milliseconds]. Note that these execution times could be improved with dedicated hardware or subsampled input sequences.

**FIGURE 6.**
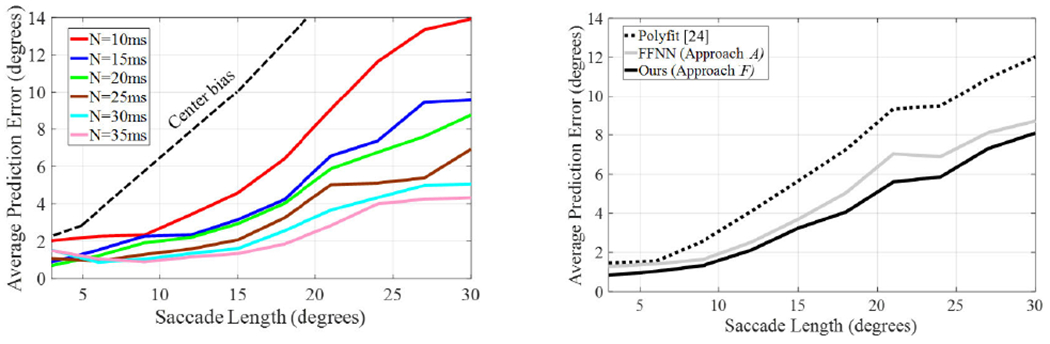
Average Prediction Error vs. Saccade Length of our approach *F* according to the number of samples (*N*) used for the prediction (Left). Comparison with previous approaches (Right). The performance of the approaches are shown as unique curves (Right) that were calculated as the average of the six curves (*N* = 10, 15,…, 35). As a baseline, center bias was obtained calculating predictions by sampling randomly from a 2D spatial Gaussian distribution whose mean and variance were determined according to the center bias model [53].

For the rest of the experiments, we used model *F* as it had a good balance between prediction accuracy and number of parameters. The prediction error was inversely proportional to the number of samples (*N*) used to make the prediction. Figure 6 (Left) shows how the performance of the proposed approach depended on *N*. The results show how the prediction error decreased when more information was available for the prediction. As described in Section 2.D, the landing point prediction was updated once new samples were available. Thus, the error decreased as the saccade progressed. This update handled the non-linear trajectories of long saccades that are common [11]. The error varied from less than 1 degree for the shortest saccades to 15 degrees for predictions made with only 10ms and the largest saccades (30 degrees). In Figure 6 (Right) we show how the proposed approach outperformed previous approaches for all saccade lengths. The fine-grained learning was better than the polynomial fitting approach [24] by more than two degrees, on average. In comparison to the FFNN proposed in [25], there was an improvement of 0.36 degrees, on average.

### C. EVALUATION OF THE EFFECTS OF COVARIATES

Landing point predictions were affected by many different factors including user-dependent (e.g. neuromotor physiognomy) and task-dependent (e.g. number of samples available to make the prediction, length of the saccade) characteristics.

**FIGURE 7.**
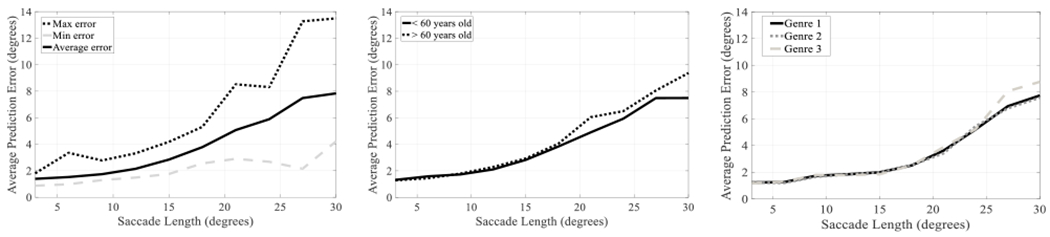
Average Prediction Error vs. Saccade Length of the best approach (approach *F*) according to the number of samples (*N*) used for the prediction. Maximum, minimum, and average Prediction Error obtained for the different users (Left). Prediction error for users younger and older than 60 years old (Center). Prediction error obtained for videos with different Genre (Genre 1 = Nature documentary, Genre 2 = Cartoon, Genre 3 = Drama) (Right). The performance of the approaches are shown as unique curves that were calculated as the average of the six curves (*N* = 10, 15,…, 35).

Some user characteristics affected the performance of the prediction. Figure 7 (Left) shows the average performance and that obtained by for best and worst participants. The large between-subject difference between the best and worst participants was significant (linear regression, t = 8.79, p < 0.001), especially for the largest saccades. We found that subject age was related to the quality of the prediction. Figure 7 (Center) shows that prediction was better for younger than older participants (shown with 60 years criterion). Presumably, older age affected the neuromotor control of the participants, and thus increasing age affected prediction performance (older subjects had slightly higher errors than younger subjects: t = 2.23, p = 0.04). Figure 7 (Right) shows that there was no difference between the genres (F(2, 20) = 0.17, p = 0.84).

Visual stimuli are the primary drivers of visual attention and thence saccades to new regions of interest. The stimuli determine the length and direction of the saccades and therefore, they affect the accuracy of the predictions. The type and salience of visual stimuli might vary between video genres. Figure 7 (Right) shows the prediction error for different types of genres (Nature Documentary, Cartoon, and Drama). Our results suggest that the genre had a limited impact on the performance of our predictions (*p*-value = 0.98). We are not claiming that visual stimuli do not affect the performance of our approach. While visual stimuli are critical to the length of the saccade, with equal length, the prediction errors were similar for the three genres.

**FIGURE 8.**
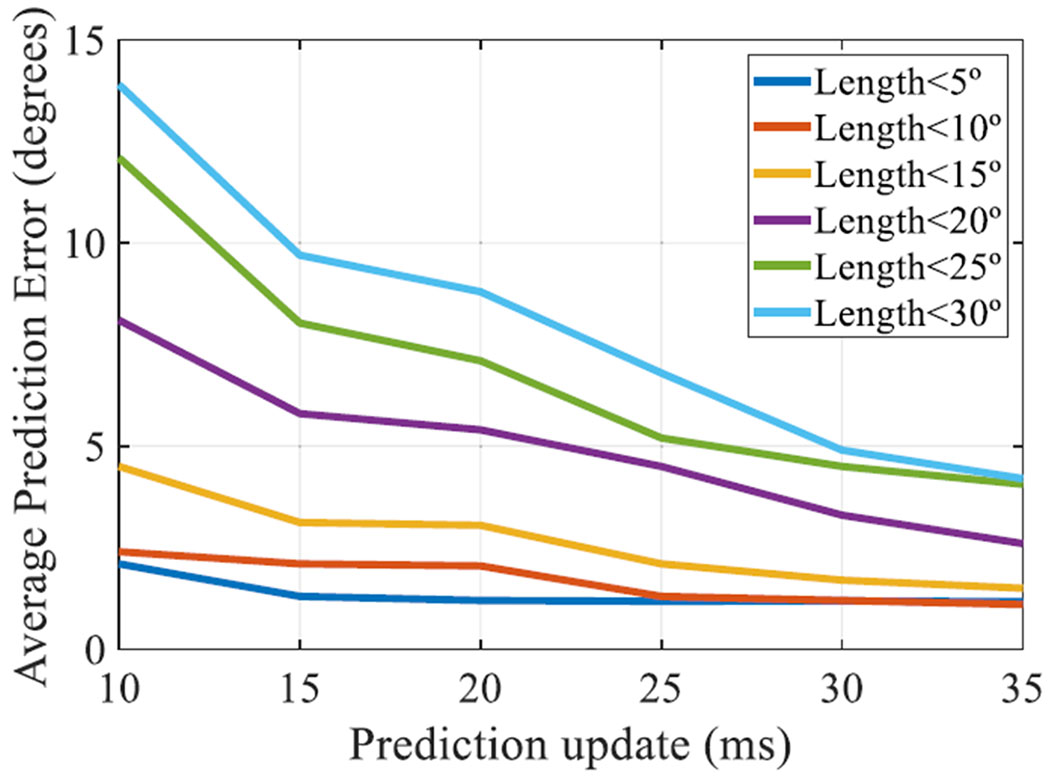
Average Prediction Error vs. Prediction update (ms), approach *F*, according to the length of the saccade (Colored image).

Figure 8 shows the performance when the landing point was updated with data provided by the eye tracker (i.e. in-flight update). In this experiment, the landing point prediction was updated every 5 milliseconds. Figure 8 shows how the predictions improved with the time and reduced the errors. This in-flight updating is an advantage of online landing prediction approaches based on eye tracking data.

**FIGURE 9.**
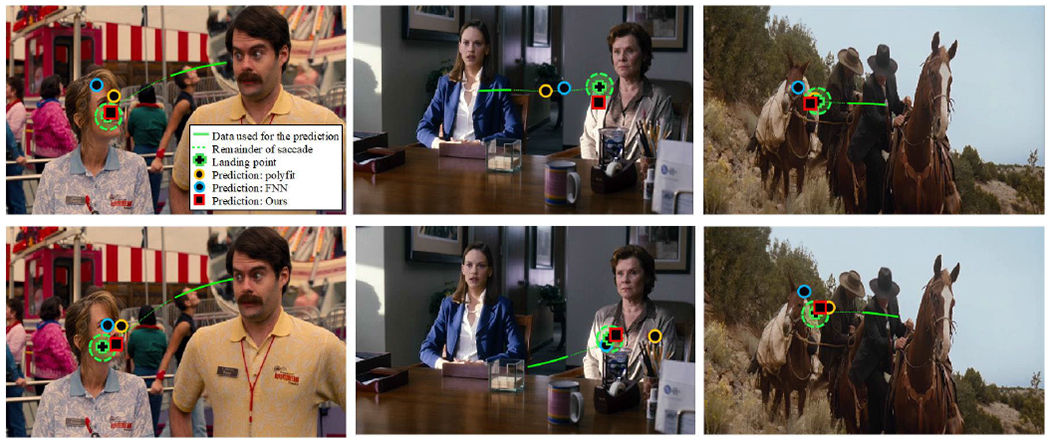
Example of the performance of the proposed method, approach *F*, and two previous approaches over real saccade trajectories. The two rows show saccades for two different participants. The trajectories have been smoothed for visualization purposes. Number of samples used for the prediction *N* = 15. The gaze positions are in the region of interest of the viewer, with the fovea represented by the green circles, each with a diameter of 2.5° (Colored image).

To illustrate the predictions of saccade-landing location, Figure 9 shows a comparison of the predictions made by three different approaches during real natural viewing for two participants watching each of three video clips. These predictions of real saccades reveal the suitability of this technology for real-world applications.

## IV. CONCLUSION

Saccades are rapid eye movements that define the region of interest of a viewer. The prediction of the saccade landing point allows a partial solution to the problems related to the update latency of gaze-contingent displays. We propose a novel algorithm to train saccade landing point predictor based on LSTM networks and fine-grained learning approach. Our strategy includes a dual loss function trained according to both gross and fine predictions. We evaluated 8 different approaches over 220,000 sequences obtained from 75 users. The data used to train and evaluate the approaches was acquired monitoring the eye movements of the participants during natural viewing of videos.

The results showed the superior performance of our proposed method with average improvement of more than two degrees in comparison with state-of-the-art methods [24], [25]. Figure 6 shows a comparison of the predictions made by different approaches during real natural viewing (Fig. 6 Right). We presented a comprehensive analysis of the performance including experiments that varied the number of samples used for the prediction (Fig. 6 Left), user-dependent effects (Fig. 7 Left), variation due to the age of the participant (Fig. 7 Center), and different video genres (Fig. 7 Right). Saccadic length and user characteristics showed the largest impact on the performance. The errors varied depending on the saccade length, with errors under three degrees for saccades smaller than 15 degrees, and errors around eight degrees for the largest saccades. Differences between participants in average prediction error was up to 9.5 degrees for the largest saccades.

Future works will include the study of user-dependent models that allow capturing the intra-user variability. The LSTM networks trained in this work could be tuned for each subject (if there was enough information from the subject) in a similar way to other domain-adaptation methods and transfer-learning techniques. The proposed learning approach can be used to predict the duration of the saccade, which is a useful information in gaze-contingent rendering. Other future research lines include the study of multimodal approaches developed to exploit both visual stimuli [12], [45]–[47] and eye tracker data [22], [24]–[26].

## Figures and Tables

**TABLE 1.** Performance of the different approaches (standard deviation in parentheses). The error rates are provided for saccades with length (E_d_) under 5 degrees, under 15 degrees, saccades larger than 15 degrees, and average error for all saccades. The errors were calculated as the average of the six errors obtained for the six models trained (*N* = 10, 15,…, 35). Bi = Bidirectional layers, T = Target, E = Average Prediction Error, ET = Averaged Execution Time in milliseconds for a single prediction (input sequence with *N* = 10 coordinates).

Approach	Ref.	Method	T	Bi	Hid. Un.	#Param*	Loss	E_d<5_	E_d<15_	E_d>15_	E_All_	ET
Baseline	[24]	Polyfit	-	-	-	18	-	1.87_(0.13)_	2.34_(0.17)_	8.43_(0.35)_	3.62_(0.21)_	0.1
*A*	[25]	FFNN	*x_M_,y_M_*	no	32×32	2K	$L_{1}$	1.39_(0.09)_	1.71_(0.11)_	6.50_(0.31)_	1.87_(0.15)_	4.5
*B*	-	RNN	*x_M_,y_M_*	no	32×32	2K	$L_{1}$	1.38_(0.09)_	1.70_(0.10)_	6.48_(0.30)_	1.85_(0.15)_	4.5
*C*	[25]	LSTM	*x_M_,y_M_*	no	32×32	13K	$L_{1}$	1.37_(0.08)_	1.68_(0.10)_	6.32_(0.30)_	1.71_(0.14)_	4.6
*D*	-	LSTM	*α,d*	no	32×32	13K	$L_{1}$	1.35_(0.09)_	1.92_(0.12)_	7.58_(0.42)_	2.34_(0.19)_	4.6
*E*	-	LSTM	*x_M_,y_M_*	no	32×32	15K	$L_{3}$	1.24_(0.06)_	1.46_(0.08)_	5.66_(0.26)_	1.54_(0.14)_	4.8
*F*	-	LSTM	*x_M_,y_M_*	no	64×64	54K	$L_{3}$	1.23_(0.07)_	1.45_(0.08)_	5.57_(0.26)_	1.51_(0.13)_	5.2
*G*	-	LSTM	*x_M_,y_M_*	no	128×128	207K	$L_{3}$	1.24_(0.7)_	1.45_(0.9)_	5.49_(0.25)_	1.52_(0.13)_	5.3
*H*	-	LSTM	*x_M_,y_M_*	yes	32×32	25K	$L_{3}$	1.29_(0.7)_	1.51_(0.10)_	5.72_(0.27)_	1.65_(0.15)_	8.8

* The number of parameters of the model is calculated for input sequences with *N*=10 coordinates.

## Footnotes

1 In this work, raw data refers to the data directly provided by the Eye Tracker.
